# Chemical composition and Biological studies of *Ficus benjamina*

**DOI:** 10.1186/1752-153X-8-12

**Published:** 2014-02-13

**Authors:** Muhammad Imran, Nasir Rasool, Komal Rizwan, Muhammad Zubair, Muhammad Riaz, Muhammad Zia-Ul-Haq, Usman Ali Rana, Ayman Nafady, Hawa ZE Jaafar

**Affiliations:** 1 Department of Chemistry, Government College University Faisalabad, Faisalabad 38000, Pakistan; 2 The Patent Office, Karachi, Pakistan; 3 Deanship of Scientific Research, College of Engineering, King Saud University, Riyadh 11421, Saudi Arabia; 4 Department of Chemistry, College of Science, King Saud University, Riyadh 1145, Saudi Arabia; 5 Department of Crop Science, Faculty of Agriculture, 43400 UPM Serdang, Selangor, Malaysia; 6 University Community Transformation Centre, Industry-Community Engagement, 43400 UPM Serdang, Selangor, Malaysia

**Keywords:** *Ficus benjamina*, Phenolic acids, HPLC analysis, Cytotoxicity, Antioxidant activity

## Abstract

**Background:**

Current study has been designed to estimate the possible antioxidant, antimicrobial and hemolytic potential of *Ficus benjamina* different parts (leaves, stem and root).

**Results:**

All examined extracts and fractions were significantly rich in antioxidants and exhibited potent antimicrobial activity. GC/MS analysis of essential oil identified four compounds in stem and eight compounds in root, respectively. HPLC analysis indicated four phenolic compounds (chlorogenic, *p*-coumaric, ferulic and syringic acids) in roots, three (chlorogenic *p*-coumaric and ferulic acids) in stem and only one (caffeic acid) in leaves. Extracts of all three parts of *F. benjamina* exhibited substantial hemolytic activity.

**Conclusions:**

Considering these results, it is concluded that *F. benjamina* can be used as a potential source for the exploration of new antioxidant compounds and antimicrobial agents.

## Background

*Ficus benjamina* L. (Moraceae), locally known as weeping fig, is a multipurpose tree found in various parts of Pakistan. *F. benjamina* is native to a large area including India, southern China, Southeast Asia, Malaysia, the Philippines, northern Australia, and the islands of the South Pacific [1]. *F. benjamina* is cultivated in many parts of the world including American Samoa (Tutuila), French Polynesia (cult.), Marshall Islands (Kwajalein (cult.), Majuro (cult.), Tonga as well as Florida, in the United State [2]. It grows as a large evergreen shrub, up to 8 m tall, with nearly 10 m wide spreading crown and drooping shoots with young slender twigs. The plant is well known due to its medicinal potential. Its latex and some fruit extracts are used by indigenous communities to treat skin disorders, inflammation, piles, vomiting, leprosy, malaria, nose-diseases and cancer besides the use as a general tonic. The plant is also used as antimicrobial, antinociceptive, antipyretic, hypotensive and anti-dysentery remedy. The leaves and twigs are used as insect repellant [3-6]. The leaves, bark and fruits of *F. benjamina* contain various bioactive constituents like cinnamic acid, lactose, naringenin, quercetin, caffeic acid and stigmasterol [5].

Despite its wide use, some literature is available about the chemistry and the biological properties of this plant [7-10]. In this context, as part of our studies on indigenous flora of Pakistan [11-21], the present study was conducted to evaluate some chemical and biological characteristics of *F. benjamina*.

## Results and discussion

### Percent yield of extracts and fractions

Determination of extract yield is an important indicator and first step during evaluation of antioxidant capacity of extracts of plants. The percent yield of different extracts and fractions (methanol extract, *n*-hexane, chloroform, ethyl acetate, *n*-butanol) of stem, root and leaves was found to be in the range of 9.7- 18 g/100 g, 8–20 g/100 g and 8.3-23.34 g/100 g, respectively. The maximum yield was exhibited by methanol extract of leaves (23.34 g/100 g). The methanol extracts of root (20 g/100 g) and stem (18 g/100 g) also showed good yield. Our results of maximum methanolic extract yield for *F. benjamina* leaves agree with those previously reported Ficus specie leaves sample (18.12%) [22]. It is believed that percentage yield of extracts depends on various factors, like plant part, season and maturity of plant part, and agroclimatic conditions from where that part is collected.

### Total phenolic contents (TPC) and total flavonoid contents (TFC)

The extract and fractions of stem, root and leaves exhibited TPC values as was measured by Folin-Ciocalteu reagent procedure in the range of 50.80-735.11, 55.02-705.11, 52.61-617.50 (Gallic acid equivalent, (GAE) mg/100 g) of dry extract, respectively. The methanol extract and *n-*butanol fractions showed greater amount of phenolic contents. The *n-*hexane fractions of all three parts exposed minimum phenolic contents with a maximum TPC value in leaves (12.61 GAE mg/100 g). The chloroform and ethyl acetate fractions indicated considerable phenolic contents, showing maximum value 263.85 (ethyl acetate) for stem (Table 1). The TPC values for methanol extracts were found to be in the range of 531–573.06 GAE mg/100 g. The *n-*butanolic fractions showed higher phenolic contents as 735.11 (GAE mg/100 g) and showed maximum values out of all fractions of three parts. The *n-*hexane fractions of all three parts showed the lowest phenolic contents. The chloroform and ethyl acetate fractions indicated considerable phenolic contents, showing maximum value 263.85 (ethyl acetate) for stem. Our results agree with earlier studies [22] that report TPC values of 100 GAE mg/100 g.

**Table 1:** **Total phenolic content, total flavonoid content and antioxidant activity of different parts of *F. benjamina***

**Antioxidant assay**	**Plant parts**			
	**Crude extracts and fractions**	**Stem**	**Root**	**Leaves**
Total phenolic contents (GAE mg/100 g)	Methanol	539.17 ± 3.21^e^	573.06 ± 2.74^d^	531.76 ± 4.90^e^
	*n*-butanol	735.11 ± 3.42^a^	705.48 ± 3.42^b^	650.17 ± 5.22^c^
	Chloroform	90.28 ± 0.11^j^	122.87 ± 0.65^i^	66.76 ± 1.07^k^
	Ethyl acetate	263.85 ± 1.71^f^	237.19 ± 1.71^g^	157.19 ± 1.71^h^
	*n*-hexane	7.80 ± 0.21l^k^	10.02 ± 0.21l^i^	12.61 ± 0.21l^l^
Total flavonoid contents (CE mg/100 g)	Methanol	724.60 ± 0.89^f^	1654.00 ± 8.93^b^	1592.20 ± 8.93^c^
	*n*-butanol	1566.40 ± 9.52^d^	1665.30 ± 9.52^b^	1780.80 ± 9.52^a^
	Chloroform	103.20 ± 2.68^k^	491.20 ± 4.55^h^	262.70 ± 2.98^j^
	Ethyl acetate	329.60 ± 4.76^i^	807.90 ± 4.76^e^	552.20 ± 4.76^g^
	*n*-hexane	4.50 ± 0.25l^j^	6.40 ± 0.12l^j^	8.40 ± 0.12l^j^
DPPH, IC_50_ (μg/ml)	Methanol	50.10 ± 3.23^i^	58.81 ± 4.50^i^	49.86 ± 3.39^i^
	*n*-butanol	152.35 ± 4.10^g^	158.44 ± 3.29^g^	147.46 ± 3.85^g^
	Chloroform	176.04 ± 4.54^f^	221.22 ± 4.20^e^	103.96 ± 2.06^h^
	Ethyl acetate	228.79 ± 5.27^de^	237.1 ± 4.69^d^	223.00 ± 2.68^e^
	*n*-hexane	554.13 ± 7.39^b^	580.75 ± 5.89^a^	436.21 ± 7.27^c^
% Inhibition in linoleic acid system	Methanol	78.16 ± 3.41^b^	85.87 ± 3.59^a^	69.81 ± 3.00^c^
	*n*-butanol	49.66 ± 2.98^ef^	81.48 ± 3.59^ab^	50.48 ± 2.47^e^
	Chloroform	16.94 ± 1.44^j^	58.52 ± 2.94^d^	42.41 ± 2.36^fg^
	Ethyl acetate	34.79 ± 2.41^h^	24.50 ± 1.88^i^	37.95 ± 1.78^gh^
	*n*-hexane	35.65 ± 2.00^gh^	20.57 ± 0.63^ij^	26.82 ± 1.85^i^

Values are means ± SD of three separate experiments (*p* < 0.05). Different letters in superscript indicate significant differences within solvents and among various parts.

The range of TFC for stem was 60.46-1466.3, for root 112.38-1554.02 and for leaves 110.45-1492.1 CE mg/100 g of dry extract, respectively. The methanol extract (1554.02) and *n-*butanolic fraction (1545.4) of root showed maximum values of flavonoid contents. The *n-*hexane fractions exhibited minimum quantity of flavonoids out of all fractions and its maximum value was 112.38. Ethyl acetate and chloroform fractions also revealed substantial values of TFC contents (Table 1). The root methanol extract and *n-*butanolic fraction showed maximum values of flavonoid contents (1554.02 and 1545.4, respectively). The *n*-hexane fractions showed lower flavonoid contents as compared to other extract and fractions. Ethyl acetate and chloroform fractions also revealed substantial values of TFC, showing maximum amount of flavonoids. However, the methanol extract of root showed maximum contents of TFC (1554.02). In addition, its *n-*butanolic fraction also disclosed tangible value (1592.1). Hence, root contained higher amounts of flavonoids than stem and leaves.

### Antioxidant activity

Free radicals are the major contributors to several clinical disorders such as diabetes mellitus, cancer, renal failures and degenerative diseases as they disturb natural defence mechanisms. These disorders can be prevented by supplementing the body’s natural antioxidant defence. Plant extracts potentiate human antioxidant defence system and are antioxidant of choice because of their lower toxicity and side effects over the synthetic ones.

Methanol extracts of stem, root and leaves exhibited IC_50_ values of 50.1, 58.81 and 49.86 μg/ml, respectively. The maximum value of IC_50_ was demonstrated by root’s fraction of *n*-hexane (580.75 μg/ml), indicating that this fraction showed minimum free radical scavenging activity. Unlike *n*-hexane fraction, chloroform and ethyl acetate fractions exhibited lower values of IC_50_ as showed in Table 1. The methanol extract and *n-*butanolic fractions showed maximum free radical scavenging activity. The *n-*hexane fractions of root revealed maximum value of IC_50_ (580.75 μg/ml). Methanol and *n-*butanol fractions exhibited the lowest IC_50_ values, showing a maximum value (158.34 μg/ml) for root.

The methanol extract and *n*-butanol fraction showed greater percent inhibition of linoleic acid system, compared to other fractions. The percent inhibition in linoleic acid system for stem wass in the range of 16.94 - 78.16%, in root 20.57-85.87% and in leaves 26.82-69.81%. The maximum percent inhibition was determined by methanol extract (85.87) and butanol fraction (81.48) of root. The results of these experiments revealed that antioxidant potential of plant increased linearly with the increase in concentration. The methanol extract as well as fractions of root exhibited a linear rise in absorbance value for various concentrations 0.56 nm: 2.5 (mg/ml), 0.87 nm: 5 (mg/ml), 1.03 nm: 7.5 (mg/ml) and 1.49 nm: 10 (mg/ml) (Table 2). The presence of phenolic compounds might be the reason for reducing power. Literature reports [23-25] indicate that the reducing power of bioactive compounds is associated with antioxidant activity. The results of this assay indicated that the plant is a good source of antioxidants with high reducing power.

**Table 2:** **Reducing potential of different parts of *F. benjamina***

**Plant extracts and fractions**	**Concentration (mg/ml)**	**Plant parts**		
		**Stem**	**Root**	**Leaves**
Methanol	2.5	0.64 ± 0.003^rs^	0.56 ± 0.003^uv^	0.39 ± 0.00^z^
	5.0	1.22 ± 0.015^e^	0.87 ± 0.001^klm^	0.57 ± 0.001^tuv^
	7.5	1.39 ± 0.006^c^	1.03 ± 0.001^g^	0.79 ± 0.001^no^
	10.0	1.47 ± 0.006^ab^	1.49 ± 0.001^a^	1.41 ± 0.003^bc^
*n*-butanol	2.5	1.02 ± 0.006^g^	0.76 ± 0.006^op^	0.38 ± 0.001^z^
	5.0	1.10 ± 0.006^f^	0.83 ± 0.001^lmn^	1.18 ± 0.006^e^
	7.5	1.22 ± 0.006^e^	0.84 ± 0.005^lmn^	1.28 ± 0.029^d^
	10.0	1.39 ± 0.026^c^	0.91 ± 0.001^ijk^	1.47 ± 0.005^ab^
Chloroform	2.5	0.37 ± 0.001^z^	0.37 ± 0.001^z^	0.64 ± 0.001^rs^
	5.0	0.39 ± 0.001^z^	0.61 ± 0.001^stu^	0.93 ± 0.001^ij^
	7.5	0.46 ± 0.001^xy^	0.62 ± 0.001st	0.94 ± 0.001^ij^
	10.0	0.47 ± 0.001^x^	0.91 ± 0.001^ijk^	0.96 ± 0.001^hi^
Ethyl acetate	2.5	0.48 ± 0.001^x^	0.60 ± 0.001^stu^	0.50 ± 0.001^wx^
	5.0	0.57 ± 0.001^tuv^	0.88 ± 0.001^jkl^	0.59 ± 0.001^sv^
	7.5	0.73 ± 0.001^pq^	1.01 ± 0.006^gh^	0.61 ± 0.001^stu^
	10.0	0.91 ± 0.001^ijk^	1.29 ± 0.023 ^d^	0.64 ± 0.001^rs^
*n*-hexane	2.5	0.48 ± 0.001^x^	0.35 ± 0.001^z^	0.68 ± 0.001^qr^
	5.0	0.49 ± 0.001^wx^	0.37 ± 0.001^z^	0.79 ± 0.003^no^
	7.5	0.54 ± 0.001^vw^	0.40 ± 0.001^yz^	0.69 ± 0.151^qr^
	10.0	0.59 ± 0.001^sv^	0.81 ± 0.001^mno^	1.12 ± 0.015^f^

Values are means ± SD of three separate experiments (*p* < 0.05). Different letters in superscript indicate significant differences within solvents and among various parts.

### Antimicrobial activity

The extracts and fractions of stem, root and leaves exhibited considerable antimicrobial activity against four bacterial and two fungal strains (Table 3). The range of antimicrobial activity expressed as diameters of inhibition zone (DIZ) for stem was 10.5 mm (*n-*hexane) - 22.83 mm (*n-*butanol). All the butanol fractions exhibited strong activity. Methanol extract (22.63 mm against *P. aeruginosa*) and *n*-butanolic fraction (22.83 against *B. subtilis*) of stem showed substantial activity. The *n*-hexane, chloroform and ethyl acetate sprouted moderate value of DIZ, with maximum value disclosed by ethyl acetate (16.88 mm). The stem extract and fractions revealed the following order of antimicrobial potential against *B. cerus*; methanolic > *n*-butanolic > ethyl acetate > chloroform > *n*-hexane.

**Table 3:** **Antimicrobial analyses of *F. benjamina *** **against bacterial and fungal strains by disc diffusion assay**

**Microbial strains**	**Plant extracts and fractions**	**Diameter of inhibition Zone in mm**		
		**Stem**	**Root**	**Leaves**
***Bacillus cerus***	Methanol	22.5 ± 0.87^b^	16.88 ± 0.36^ef^	12.00 ± 0.29^gh^
	*n*-butanol	22.25 ± 0.72^b^	17.73 ± 0.16^e^	19.50 ± 0.29^cd^
	Chloroform	12 ± 0.58^gh^	15.63 ± 0.22^f^	10.75 ± 0.14^hi^
	Ethyl acetate	17 ± 0.58^ef^	16.88 ± 0.07^ef^	10.00 ± 0.58^i^
	*n*-hexane	11.25 ± 0.72^hi^	12.99 ± 0.02^g^	6.50 ± 0.29^j^
	Ampiciline	24.6 ± 0.66^a^	18.25 ± 0.43d^e^	20.75 ± 0.72^c^
***Pseudomonas aerugonisa***	Methanol	23.5 ± 0.29^a^	16.75 ± 0.43d^e^	12.04 ± 0.31^fg^
	*n*-butanol	22 ± 0.87^b^	17.23 ± 0.13^d^	19.13 ± 0.07^c^
	Chloroform	12.25 ± 0.72^fg^	15.88 ± 0.36^e^	10.5 ± 0.29^h^
	Ethyl acetate	16.75 ± 0.72^de^	17.13 ± 0.22d^e^	10.25 ± 0.43^h^
	*n*-hexane	11 ± 0.58^gh^	13.09 ± 0.10^f^	7.50 ± 0.29^i^
	Ampiciline	24.38 ± 0.79^a^	17.75 ± 0.14^d^	20.25 ± 0.43^c^
***Escherichia coli***	Methanol	22.63 ± 0.22^b^	16.47 ± 0.59^ef^	11.75 ± 0.14^h^
	*n*-butanol	22.75 ± 0.43^b^	16.23 ± 0.71^ef^	18.25 ± 0.43^c^
	Chloroform	11.57 ± 0.33^h^	15.63 ± 0.22^f^	11.58 ± 0.33^h^
	Ethyl acetate	16.88 ± 0.65^de^	16.88 ± 0.07^de^	10.00 ± 0.58^i^
	*n*-hexane	10.61 ± 0.35^hi^	13.34 ± 0.24^g^	6.50 ± 0.29^j^
	Ampiciline	24.65 ± 0.64^a^	17.87 ± 0.71^ef^	18.75 ± 0.43^c^
***Bacillus subtilis***	Methanol	22.5 ± 0.29^b^	16.43 ± 0.62^e^	12.08 ± 0.29^f^
	*n*-butanol	22.83 ± 0.39^b^	16.34 ± 0.64^e^	18.50 ± 0.29^c^
	Chloroform	12 ± 0.58^f^	15.75 ± 0.29^e^	12.13 ± 0.65^f^
	Ethyl acetate	16.63 ± 0.79^de^	16.5 ± 0.14^de^	10.50 ± 0.29^g^
	*n*-hexane	10.5 ± 0.29^g^	12.96 ± 0.02^f^	7.13 ± 0.07^h^
	Ampiciline	24.45 ± 0.75^a^	17.75 ± 0.14^cd^	20 ± 0.51^c^
***Aspergillus niger***	Methanol	19.25 ± 0.43^c^	14.60 ± 0.23^e^	12.11 ± 0.29^f^
	*n*-butanol	21.13 ± 0.51^b^	14.98 ± 0.27^e^	19.50 ± 0.29^c^
	Chloroform	11.7 ± 0.40^f^	17.15 ± 0.66^d^	10.38 ± 0.36^g^
	Ethyl acetate	16.62 ± 0.22^d^	16.38 ± 0.22^d^	10.50 ± 0.29^g^
	*n*-hexane	10.25 ± 0.14^g^	14.70 ± 0.40^e^	7.63 ± 0.36^h^
	Terbinafine	24.38 ± 0.79^a^	16.75 ± 0.43^d^	20.25 ± 0.43b^c^
***Candida albicans***	Methanol	18.75 ± 0.72^de^	14.88 ± 0.07^h^	12.15 ± 0.37^i^
	*n*-butanol	21.38 ± 0.36^b^	15.23 ± 0.13^h^	19.75 ± 0.43^cd^
	Chloroform	11.88 ± 0.51^ij^	17.44 ± 0.83^ef^	10.62 ± 0.22^jk^
	Ethyl acetate	16.88 ± 0.07^fg^	16.13 ± 0.36^gh^	10.38 ± 0.36^k^
	*n*-hexane	10.63 ± 0.36^jk^	14.95 ± 0.55^h^	7.88 ± 0.51^l^
	Terbinafine	24.5 ± 0.72^a^	16.92 ± 0.33^fg^	20.25 ± 0.43^bc^

Values are means ± SD of three separate experiments (*p* < 0.05). Letters in superscript show the significance of the results against a single strain and among various parts.

Stem extract and fractions exhibited a different order against the two fungal strains tested. Root extract and fractions disclosed DIZ values in the range of 12.96 mm (*n*-hexane) to 17.73 mm (*n*-butanol) against bacterial strains. The maximum value of DIZ was exhibited by methanol extract and *n*-butanolic fraction. The chloroform and ethyl acetate fractions of root also showed good values. Methanol, chloroform, ethyl acetate and *n*-butanolic fractions of root exhibited moderate activity with maximum activity given by *n*-butanolic fraction against *B. cereus*. Overall, *n*-hexane faction of root showed poor activity.

In case of fungal strains, chloroform and ethyl acetate fractions of root evinced higher antimicrobial potential as compared to methanol and *n*-butanolic fractions. The root extract and fractions of methanol, *n*-butanol, chloroform, ethyl acetate and *n*-hexane exhibited the following values of DIZ against *A. niger*: 14.60, 14.98, 17.15, 16.38, 14.70 mm, respectively. The butanol fraction from leaves again showed higher antimicrobial potential as is evident from its values 19.50 mm and 19.75 mm against *B. cerus* and *C. albicans,* respectively. The methanol, *n*-hexane, chloroform and ethyl acetate fractions of leaves showed moderate activity with considerable value shown by chloroform fraction (12 mm). However, *n*-butanol fraction of leaves showed strong activity against *B. cerus*. Comparatively, *n*-hexane and ethyl acetate fractions denoted lower antimicrobial potential. Results of present study indicate that *F. benjamina* extracts can be used as a potential antimicrobial agent to inhibit the growth of various dangerous microbes.

The results of minimum inhibitory concentration (MIC) are presented in Table 4. The MIC values were inverse to antimicrobial values; the butanolic fraction of stem showed the maximum antimicrobial. The range of its MIC values was found to be 0.65 -1.79 mg/ml, indicating that it might act as an antimicrobial at low concentrations. Similarly, *n-*butanolic fractions of root and leaves exhibited maximum antimicrobial activity with MIC of 0.77-1.99 mg/ml for root and 0.7-1.5 mg/ml for leaves, respectively. Similar results were showed by the MIC analyses of fungal strains (Table 4).

**Table 4:** **Minimum inhibitory concentration (mg/ml) of *F. benjamina *** **against bacterial and fungal strains**

**Microbial strains**	**Plant extracts and fractions**	**MIC as mg/ml**		
		**Stem**	**Root**	**Leaves**
***Bacillus Cerus***	Methanol	1.03 ± 0.03^f^	0.96 ± 0.04^f^	1.02 ± 0.02^f^
	*n*-butanol	1.79 ± 0.15^bc^	1.49 ± 0.02^de^	1.54 ± 0.02^cde^
	Chloroform	2.16 ± 0.30^a^	1.75 ± 0.01^bcd^	1.74 ± 0.02^bcd^
	Ethyl acetate	1.78 ± 0.15^bc^	1.92 ± 0.03^ab^	0.99 ± 0.01^f^
	*n*-hexane	1.41 ± 0.15^e^	1.54 ± 0.03^cde^	1.59 ± 0.01^cde^
	Ampiciline	1.03 ± 0.05^f^	0.5 ± 0.01^g^	0.46 ± 0.03^g^
***Pseudomonas aerugonisa***	Methanol	2.11 ± 0.10^cd^	2.01 ± 0.01^cde^	0.54 ± 0.02^gh^
	*n*-butanol	0.83 ± 0.06^g^	2.97 ± 0.05^a^	0.77 ± 0.01^gh^
	Chloroform	2.49 ± 0.38^b^	1.75 ± 0.03^ef^	0.86 ± 0.03^g^
	Ethyl acetate	2.18 ± 0.19b^c^	1.88 ± 0.06^cde^	1.95 ± 0.03^cde^
	*n*-hexane	3.10 ± 0.08^a^	1.70 ± 0.06^ef^	1.55 ± 0.03^f^
	Ampiciline	1.85 ± 0.06^de^	0.48 ± 0.01^h^	0.48 ± 0.01^h^
***Escherichia coli***	Methanol	1.94 ± 0.07^c^	0.26 ± 0.01^i^	0.28 ± 0.02^i^
	*n*-butanol	0.88 ± 0.07^d^	0.76 ± 0.01^def^	0.73 ± 0.02^d-g^
	Chloroform	3.16 ± 0.16^b^	0.88 ± 0.01^d^	0.47 ± 0.02^ghi^
	1Ethyl acetate	`	0.94 ± 0.03^d^	0.52 ± 0.02^f-i^
	*n*-hexane	6.51 ± 0.29^a^	0.81 ± 0.01^de^	0.42 ± 0.02^hi^
	Ampiciline	0.34 ± 0.06^hi^	0.27 ± 0.01^i^	0.26 ± 0.01^i^
***Bacillus subtilis***	Methanol	1.84 ± 0.13^b^	0.58 ± 0.04^hij^	0.55 ±0.02^hij^
	*n*-butanol	0.65 ± 0.06^gh^	0.78 ± 0.02^fgh^	1.5 ± 0.01^cd^
	Chloroform	1.25 ± 0.31^de^	3.44 ± 0.05^a^	1.68 ± 0.02^bc^
	Ethyl acetate	0.95 ± 0.04^ef^	1.92 ± 0.07^b^	0.95 ± 0.04^efg^
	*n*-hexane	3.42 ± 0.30^a^	3.18 ± 0.09^a^	1.61 ± 0.02^bc^
	Ampiciline	0.41 ± 0.09^ij^	0.99 ± 0.01^ef^	0.28 ± 0.0^1j^
***Aspergillus niger***	Methanol	0.52 ± 0.006^f^	0.27 ± 0.006^i^	0.52 ± 0.02^fg^
	*n*-butanol	0.74 ± 0.006^e^	0.77 ± 0.009^de^	0.81 ± 0.03^cd^
	Chloroform	0.9 ± 0.006^c^	0.84 ± 0.023^c^	0.46 ± 0.02^h^
	Ethyl acetate	0.98 ± 0.006^a^	0.54 ± 0.021^f^	0.94 ± 0.03^a^
	*n*-hexane	0.83 ± 0.006^c^	0.84 ± 0.015^c^	0.77 ±0.01^de^
	Terbinafine	0.49 ± 0.006^g^	0.28 ± 0.015^i^	0.27 ± 0.02^i^
***Candida albicans***	Methanol	0.52 ± 0.009^f^	0.27 ± 0.009^i^	0.51 ± 0.023^f^
	*n*-butanol	0.73 ± 0.006^e^	0.77 ± 0.012^d^	0.81 ± 0.035^c^
	Chloroform	0.89 ± 0.019^b^	0.84 ± 0.019^c^	0.45 ±0.009^h^
	Ethyl acetate	0.97 ± 0.006^a^	0.54 ± 0.024^f^	0.94 ± 0.020^a^
	*n*-hexane	0.83 ± 0.009^c^	0.84 ± 0.019^c^	0.77 ± 0.015^d^
	Terbinafine	0.49 ± 0.007^g^	0.27 ± 0.018^i^	0.27 ± 0.012^i^

Values are means ± SD of three separate experiments (*p* < 0.05). Letters in superscript show the significance of the results against a single strain and among various parts. Ampiciline and Terbinafine were used as standards for bacteria and fungi, respectively.

### HPLC analysis of phenolic acids

The HPLC analysis for the presence of phenolic acids permitted the identification of 5 phenolic acids, three in stem, four in root and one in leaves. Results are presented in Table 5. Sirisha and co-workers [5] reported the presence of ursolic, α-hydroxy ursolic, protocatechuic and maslinic acids in *Ficus* species, while cinnamic and caffeic acids and quercetin have been reported in leaves, bark and fruits of *F. benjamina*[6]. All the detected phenolic acids are known to have antimicrobial and antioxidant properties [26-28]. So these phenolic acids may be responsible for antimicrobial and antioxidant activities of *Ficus benjamina.*

**Table 5:** HPLC analysis of stem, root and leaves for phenolic acids

**Phenolic acids**	**Stem (ppm)**	**Root (ppm)**	**Leaves (ppm)**
Chlorogenic acid	0.936	5.538	ND*
Paracoumaric acid	0.11	0.13	ND
Ferulic acid	2.88	1.29	ND
Syringic acid	ND	0.54	ND
Caffeic acid	ND	ND	47.61

*ND = not detected.

### Evaluation of hemolytic activity

Table 6 reports the hemolytic activity of different extracts and fractions from *F. benjamina.* The maximum hemolytic activity was shown by chloroform fractions of stem (3.36%) and leaves (3.29%). The mechanical stability of the erythrocytic membrane is a good indicator of cytotoxity. The percentage lysis of human erythrocytes was below 5.0% for all samples, so it can be expected that the extract and fractions have a no cytotoxity [29,30].

**Table 6:** **Percent haemolysis caused by *F. benjamina***

**Plant extract and fraction**	**Stem**	**Root**	**Leaves**
Methanol	1.54 ± 0.023	1.89 ± 0.061	1.93 ± 0.241
*n*-butanol	2.49 ± 0.061	1.64 ± 0.040	1.13 ± 0.023
Chloroform	3.36 ± 0.080	1.46 ± 0.061	3.29 ± 0.061
Ethyl acetate	2.26 ± 0.061	1.81 ± 0.100	2.09 ± 0.061
*n*-hexane	2.91 ± 0.046	0.90 ± 0.100	1.92 ± 0.105
Triton	99.8 ± 0.612	99.8 ± 0.612	99.8 ± 0.612
Phosphate buffer saline	0	0	0

Values are means ± SD of three separate experiments (*p* < 0.05).

### GC-MS analysis

Nine chemical constituents have been identified from essential oil of stem and root of *F. benjamina* obtained by hydrodistillation and subsequently subjected to GC-MS analysis. Stem essential oil contained four compounds: 2-pentanone, hexadecanoic acid, palmitic acid, 9,12-octadecadienoic acid; roots contained eight compounds: methanamine, cyclopentanone, methyl-2 phenylindole, cyclopropaneoctanal, arsenous acid, hexadecanoic acid, palmitic acid and 9,12-octadecadienoic acid (Table 7).

**Table 7:** **Chemical composition of *F. benjamina *** **(Stem, Root) essential oils**

**Retention time (min)**	**Chemical constituents**	**% Area**	
		**Stem**	**Root**
2.17	2-Pentanone	0.1	n.d
10.26	Hexadecanoic acid	0.34	1.98
11.04	Palmitic acid	0.84	1.89
11.91	9,12-Octadecadienoic acid	0.47	3.55
0.16	Methanamine	n.d	1.62
2.297	Cyclopentanone	n.d	0.12
10.63	Methyl-2 Phenylindole	n.d	0.12
12.53	Cyclopropaneoctanal	n.d	0.44
15.73	Arsenous acid	n.d	0.15

## Materials and methods

### Collection of plant material

The stem, root and leaves of *F. benjamina* were collected from the Botanical Garden, University of Agriculture, Faisalabad, Pakistan and were further authenticated by the Taxonomist, Dr. Mansoor Hameed, Department of Botany, University of Agriculture, Faisalabad, Pakistan where a voucher specimen has been deposited.

### Preparation of extracts and fractions

The stem, root and leaves of *F. benjamina* were washed with cold water to remove dust and other extraneous matter. The shaded dried parts of plant were grinded into powder form (500 g) and extracted with methanol at room temperature. The methanol extracts were concentrated with rotary evaporator. Then the methanol extracts of stem (80 g), root (80 g) and leaves (80 g) were further fractioned with different polarity based solvents. The stem methanolic extract was suspended in distilled water and fractioned with *n*-hexane (10 g), chloroform (8 g), ethyl acetate (9.7 g) and *n*-butanol (12 g). Root methanolic extract give the following fractions: *n*-hexane, 9 g; chloroform, 8 g; ethyl acetate, 12 g: *n*-butanol, 13 g. Leaves methanol extract was fractionated and *n*-hexane (12.4 g), chloroform (10 g), ethyl acetate (8.3 g) and *n*-butanol (10 g) fractions were obtained.

### Antibacterial and Antifungal assay

#### Test microorganisms

*Aspergillus niger* ATCC 10595*, Candida albicans* ATCC 32612 were used as the fungal tested organisms and *Pseudomonas aerugonisa* locally isolated*, Escherichia coli* ATCC 25922*, Bacillus subtilis* JS 2004*, Bacillus cerus* locally isolated were used as the bacterial tested organisms. The pure bacterial and fungal strains were obtained from the Department of Veterinary Microbiology, University of Agriculture, Faisalabad, Pakistan. The bacterial strains were cultured overnight at 37°C in nutrient agar (Oxoid, Hampshire, UK) while fungal strains were cultured overnight at 28°C using potato dextrose agar (Oxoid).

#### Antibacterial and antifungal assay by disc diffusion method

Antimicrobial activity was determined by using the disc diffusion method [21]. All samples (dry residue) were dissolved in 10% sterile dimethyl sulfoxide. The discs (6 mm diameter) were impregnated with 10 mg/mL extract/fractions (100 μL/disc) placed aseptically on the inoculated agar. Discs injected with 100 μL of respective solvents served as a negative controls, rifampcin (100 μL/disc) (Oxoid) and fluconazole (100 μL/disc) (Oxoid) were used as positive reference for bacteria and fungi, respectively. The Petri dishes were incubated at 37 ± 0.1°C for 20–24 h and 28 ± 0.3°C for 40–48 h for bacteria and fungi, respectively. At the end of period, the inhibition zones formed on the media were measured. The positive antimicrobial activity was read based on growth inhibition zone.

#### Minimum inhibitory concentrations (MIC) of plant extracts

Minimum inhibitory concentration both for bacterial and fungal strains was measured as reported in literature [31].

### Determination of total phenolic contents

Amount of total phenolic contents were assessed using Folin-Ciocalteu reagent procedure [32]. Briefly, 1 mg of dry mass of crude extracts/fractions was mixed with 0.5 mL of Folin-Ciocalteu reagent and 7.5 mL deionized water. The mixture was kept at room temperature for 10 min and then 1.5 mL of 20% sodium carbonate (w/v) was added. The mixture was heated in a water bath at 40°C for 20 min and then cooled in an ice bath; finally absorbance at 755 nm was measured (Hitachi U-2001 spectrophotometer). Amounts of TP were calculated using a calibration curve for gallic acid (10–100 ppm) (R2 = 0.9986). The results were expressed as gallic acid equivalents (GAE) of dry plant matter.

### Determination of total flavonoid contents

The total flavonoid content in plant extract and fractions was determined by following the already reported procedure [32]. Plant extract/fractions of each material (1 mL containing 0.1 g/mL) was placed in a 10 mL volumetric flask, then added distilled water 5 mL and 0.3 mL of 5% NaNO_2_ was added to each volumetric flask initially; after 5 min, 0.6 mL of 10% AlCl_3_ was added. After another 5 min, 2 mL of 1 M NaOH was added and volume made up with distilled water. Then solution was mixed. At 510 nm absorbance of the reaction mixture was taken using a spectrophotometer. Total flavonoid content were evaluated as catechin equivalents (CE g/100 g of dry plant matter).

### Antioxidant analysis

#### Measurement of reducing potential

The reducing potential of plant extract was determined by methods reported in literature [33].

Equivalent volume of plant crude extracts/fractions containing 2.5-10 mg of dry matter was mixed with sodium phosphate buffer (5.0 mL, 0.2 M, pH 6.6) and potassium ferricyanide (5.0 mL, 1.0%); the mixture was incubated at 50°C for 20 min. Then 5 mL of 10% trichloroacetic acid was added and centrifuged at 980 xg for 10 min at 5°C in a refrigerated centrifuge. The upper layer of the solution (5.0 mL) was diluted with 5.0 mL of distilled water and ferric chloride (1.0 mL, 0.1%) and absorbance noted at 700 nm (Hitachi U-2001 spectrophotometer). The measurement was run in triplicate and results averaged.

#### DPPH free radical scavenging assay

The 2,2-diphenyl-1-picrylhydrazyl radical (DPPH) assay was carried out spectrophotometrically as described by Bozin and coworkers [34]. Aliquots (50 μL) of various concentrations (10–100 μg/mL) of the plant extract/fractions was added to 5 mL of a 0.004% methanolic solution of DPPH. After 0.5 h incubation period at room temperature, the absorbance was read against a blank at 517 nm:

$$\mathrm{Inhibition}\;\left(\%\right)=100\times \left(A\mathrm{blank}-A\mathrm{sample}/A\mathrm{blank}\right)$$

where *A*blank is the absorbance of the control reaction (containing all reagents except the test compound) and *A*sample is the absorbance of the test compound. Extract/fraction concentration providing 50% inhibition (IC50) was calculated from a graph plotting percentage inhibition against extract concentration.

#### Antioxidant activity determination in linoleic acid system

The antioxidant activity was also determined in terms of measurement of percent inhibition of peroxidation in linoleic acid system, as previously reported [35]. Extracts/frations (5 mg) were added to a solution mixture of linoleic acid (0.13 mL), 99.8% ethanol (10 mL) and 10 mL of 0.2 M sodium phosphate buffer (pH = 7.4). Total mixture was diluted to 25 mL with distilled water. The solution was incubated at 40°C and the degree of oxidation was measured following thiocyanate method with 10 mL of ethanol (75%), 0.2 mL of an aqueous solution of ammonium thiocyanate (30%), 0.2 mL of sample solution and 0.2 mL of ferrous chloride (FeCl2) solution (20 mM in 3.5% HCl) being added sequentially. After 3 min of stirring, the absorption values of mixtures measured at 500 nm were taken as peroxide contents. A control was performed with linoleic acid but without extracts. Synthetic antioxidants; butylated hydroxytoluene (BHT) was used as positive control. The maximum peroxidation level observed as 360 h (15 days) in the sample that contained no antioxidant component was used as a test point.

Percent inhibition of linoleic acid peroxidation = 100 – [(Abs. increase of sample at 360 h/abs. increase of control at 360 h) x100], was calculated to express antioxidant activity.

### Hemolytic activity

Hemolytic activity was checked by following the reported method [29]. Briefly, three ml of freshly obtained heparinzed human blood was gently mixed, poured into sterile 15 ml falcon tube and centrifuged for 5 min at 850 × g. The supernatant was poured off and viscous pellet washed three additional times with 5 ml of chilled (4°C) sterile isotonic phosphate-buffered saline solution (PBS) solution, adjusted to pH = 7.4. The washed cells were suspended in the 20 ml chilled, saline PBS buffer. Erythrocytes were counted on a haemacytometer (Fisher ultra plane Neubauer ruling). Twenty μl of plant extract (15 mg/ml) in five different solvent was taken in 20 ml Eppendorff tubes. For each assay, 0.1% Triton X-100 was taken as a positive control, 100% blood lysis and Phosphate buffer saline (PBS) was taken for each assay as a negative control (0% lysis). To each 2 ml Eppendorff tube already containing 20 μl sample, 180 μl diluted blood cell suspension was added and mixed. Tubes were incubated for 35 min at 37°C and agitate after 10 min. Immediately after incubation, the tubes were placed on ice for 5 min and then centrifuged for 5 min at 1310xg. After centrifugation, 100 μl supernatant was taken from the tubes and diluted with 90 μl chilled PBS. Then, 200 μl were placed into 96 well plates. After this, absorbance at 576 nm was measured. Triton-X (0.1%) was taken as a positive control for 100% lyses and PBS buffer as negative control for 0% lyses. The experiment was done in triplicate and mean ± S.E. was calculated using the following formula:

$$\mathrm{percent}\;\mathrm{hemolysis}=\left(\mathrm{Hb}\;\mathrm{ABS}/\mathrm{Hb}100\right)\times 100$$

### Sample extraction for HPLC analysis

The methanolic extracts of stem root and leaves were prepared by using 0.5 g of dry-ground sample and 20 mL of solvent. The mixtures were shaken in a c24k refrigerated incubator shaker (NJ, USA) at room temperature for 60 min. Then, the mixtures were centrifuged in a Universal 320r Hettich Zenrifugen (Tuttlingen, Germany) at 9000 rpm for 5 min. at 4°C. The supernatant was recovered and used for the determination of phenolic acids by HPLC [36]. The supernatants were filtered through a 45 μm filter prior to analysis. All the extractions were performed in duplicate and the supernatants were kept at -20°C until further analysis.

An HPLC (model LC-10A, Shimadzu, Japan) equipped with two LC-10 AS pumps, SCL-10A system control unit, Rheodyne injector, CTO-10A column oven, SPD-10A UV–vis detector and data acquisition class CSW32 soft ware was used.

### Gas Chromatography/Mass Spectrometry analysis

The GC-MS analysis of the essential oils was carried out using a GC 6850 Network gas chromatographic system, equipped with a 7683 B Series auto injector and a 5973 I inert mass selective detector (Agilent Technologies USA), using a HP-5 MS capillary column having 5% phenyl polysiloxane as a stationary phase, column length 30.0 m, i.d. 0.25 mm and film thickness 0.25 μm. One μl of sample was injected in the split mode with split ratio 30:1 at a temperature of 300°C. Helium was used as carrier gas with a constant flow rate of 1.5 ml/min. The temperature program was as follows: initial temperature 150°C, held for 1 min and then ramping at rate of 10°C/min up to 290°C, kept constant for 5 min. The temperature of transfer line was 300°C. Electron ionization mode with the ionization energy of 70 eV having mass range scanned 3–500 m/z was used for mass spectra determination. The temperature of ion source was 230°C and that of MS quadropole 150°C. The identification of components was based on comparison of their mass spectra with those of NIST mass spectra library [37,38].

### Statistical analysis

Two factor completely randomized design (CRD) was applied and significant difference among means was worked out using Duncan’s multiple range (DMR) test at 5% level of significance.

## Conclusion

Present study ascertained the potency of the *F. benjamina* as a potential source of natural antioxidants and antimicrobial agents. The root and leaves showed good antioxidant activity, whereas stem extract and fractions revealed good antimicrobial activity. *Ficus benjamina* disclosed substantial bioactivity, being root extract and fractions the most active. This plant can be regarded as a potential source of antioxidant and antimicrobial agents.

## Competing interests

The authors declare that they have no competing interests.

## Authors’ contributions

MI, NR, KR, MZ, UAR, AN, and MR made a significant contribution to experiment design, acquisition of data, analysis and drafting of the manuscript. MZUH and HZEJ have made a substantial contribution to interpretation of data, drafting and carefully revising the manuscript for intellectual content. All authors read and approved the final manuscript.
